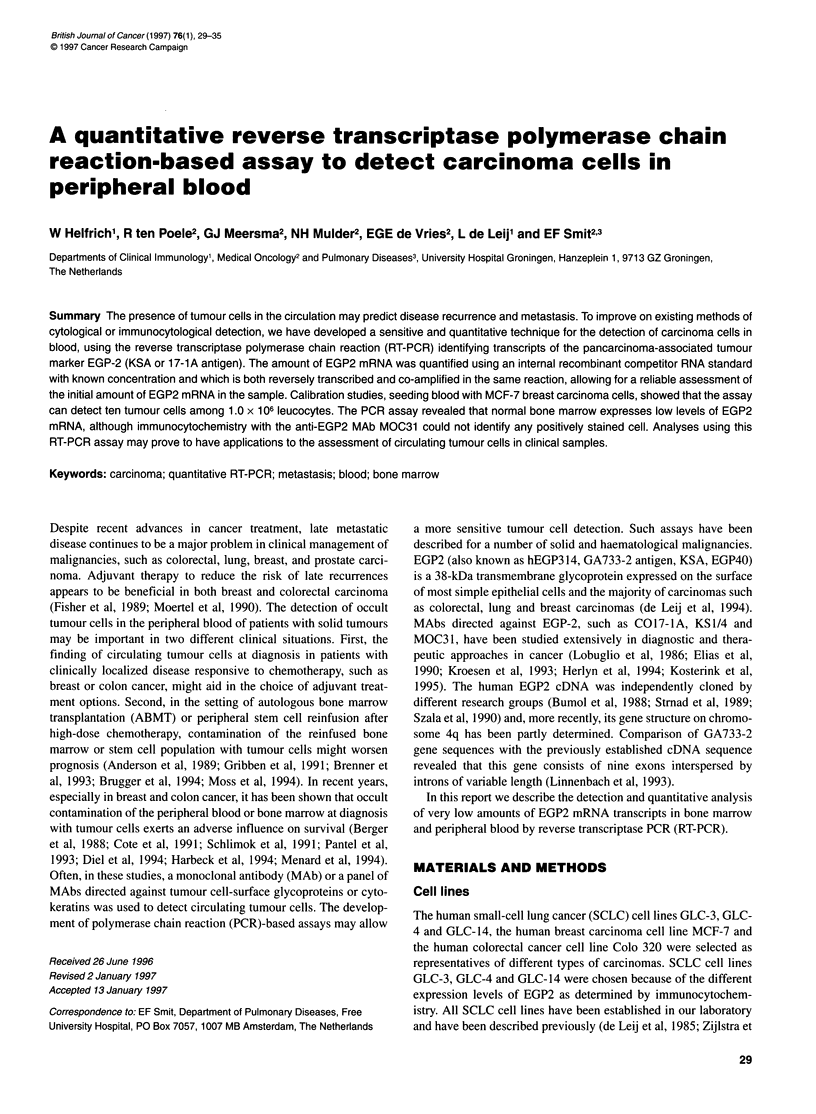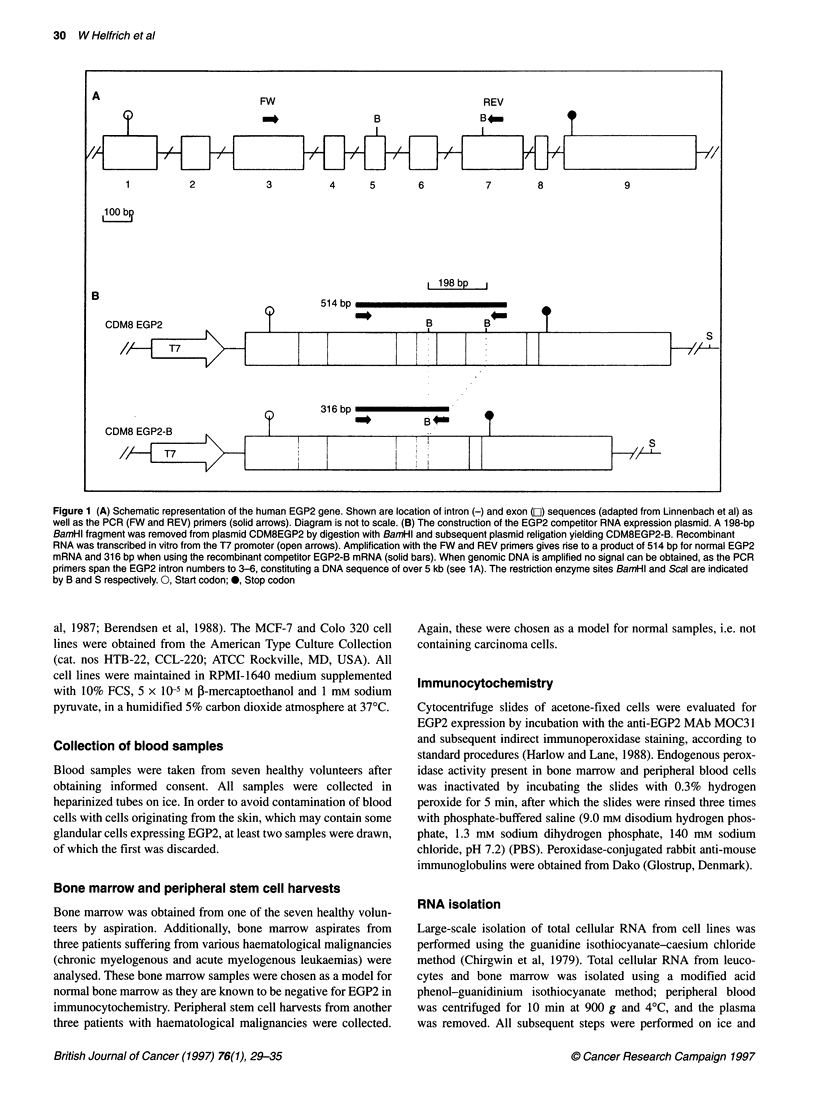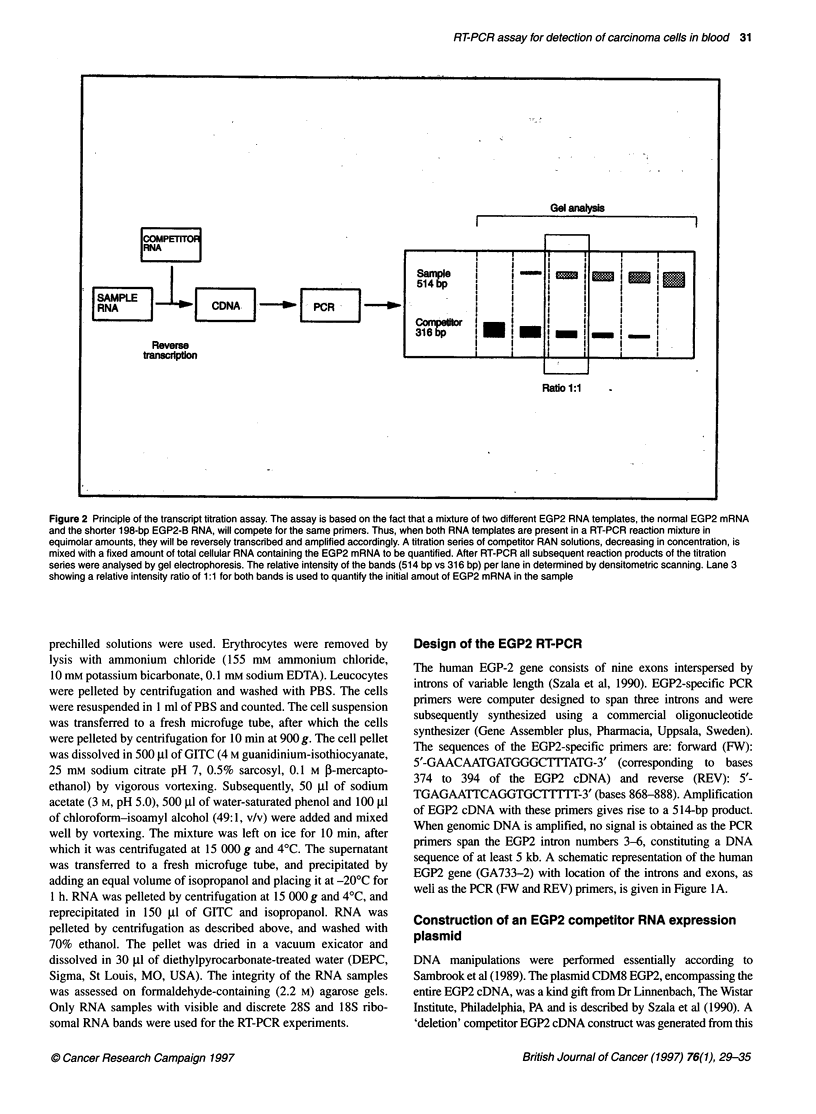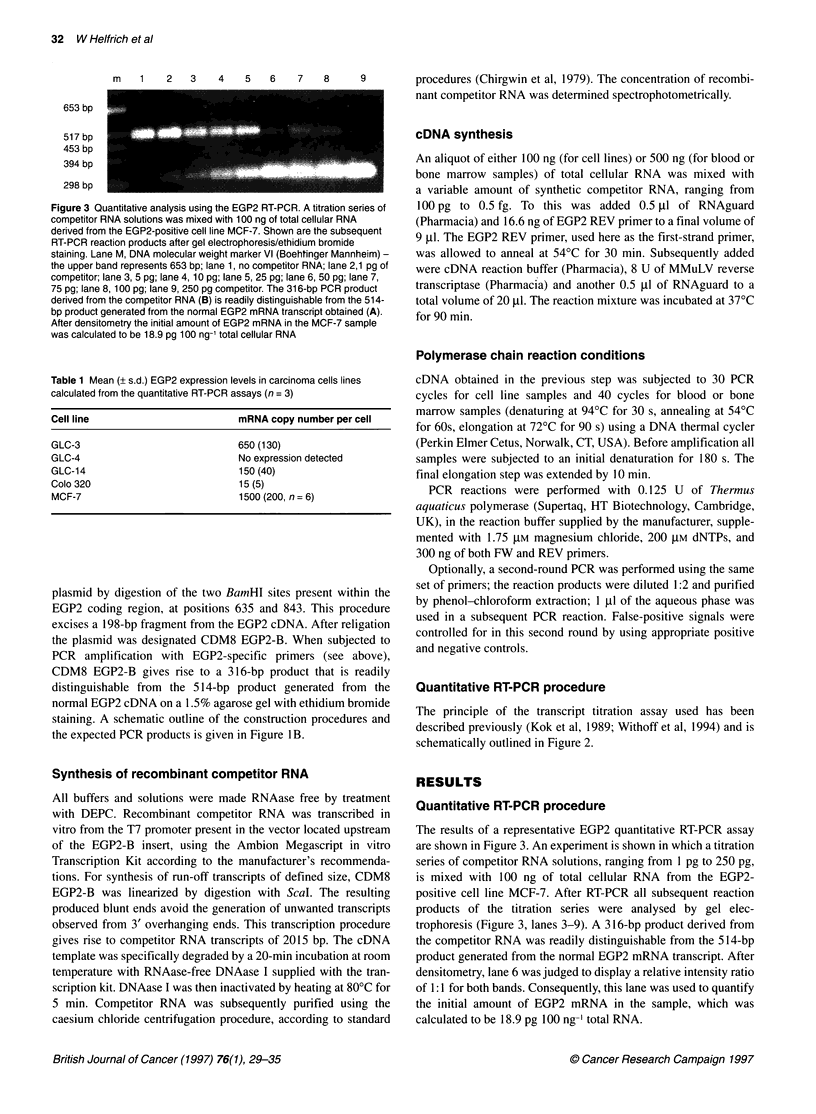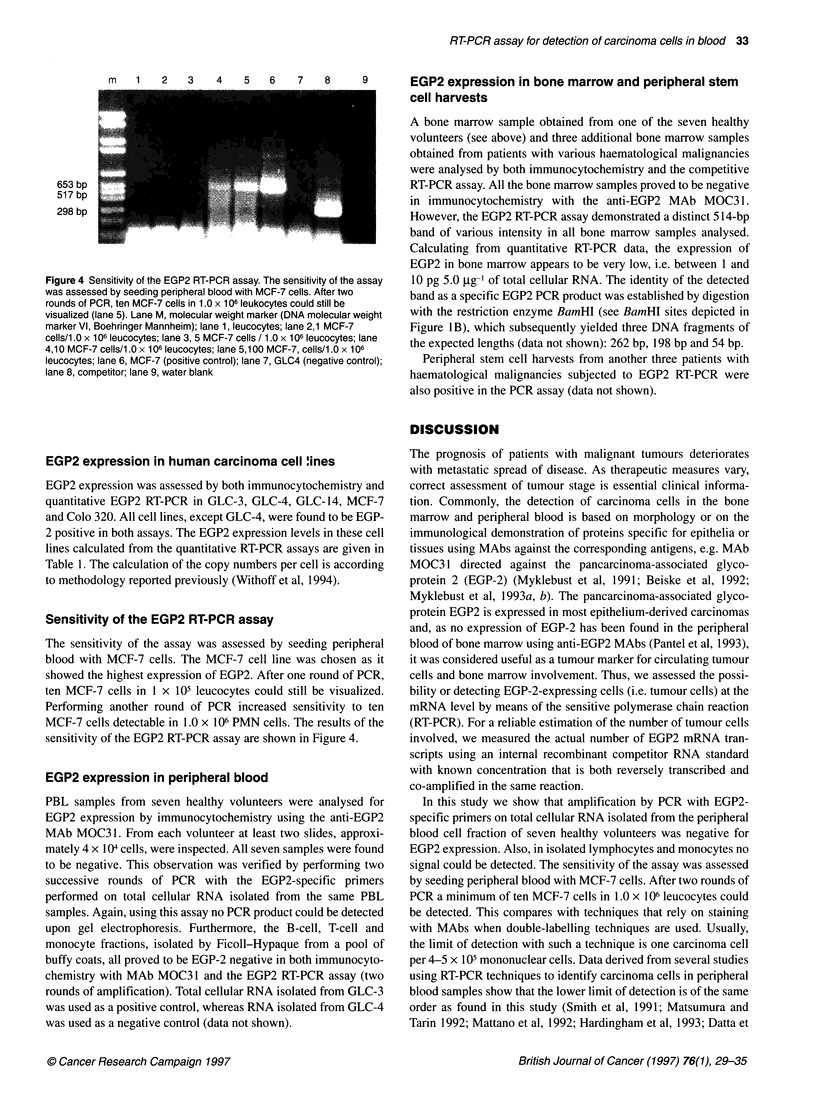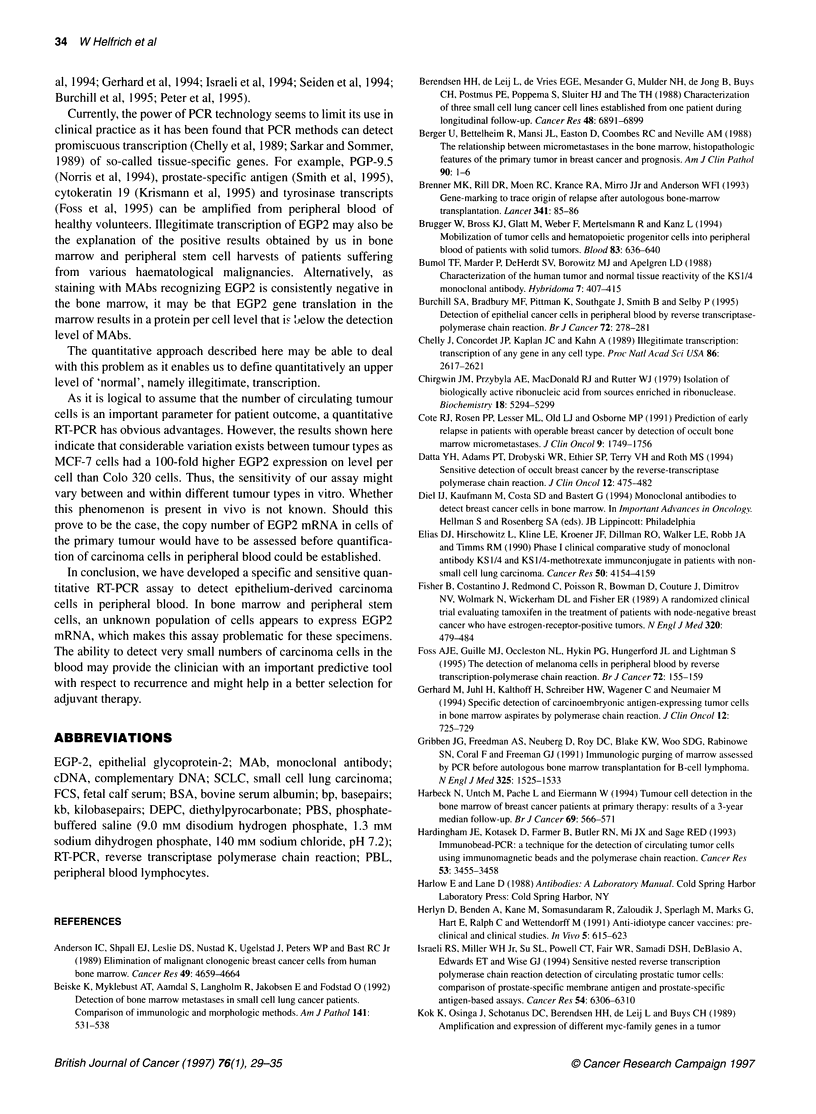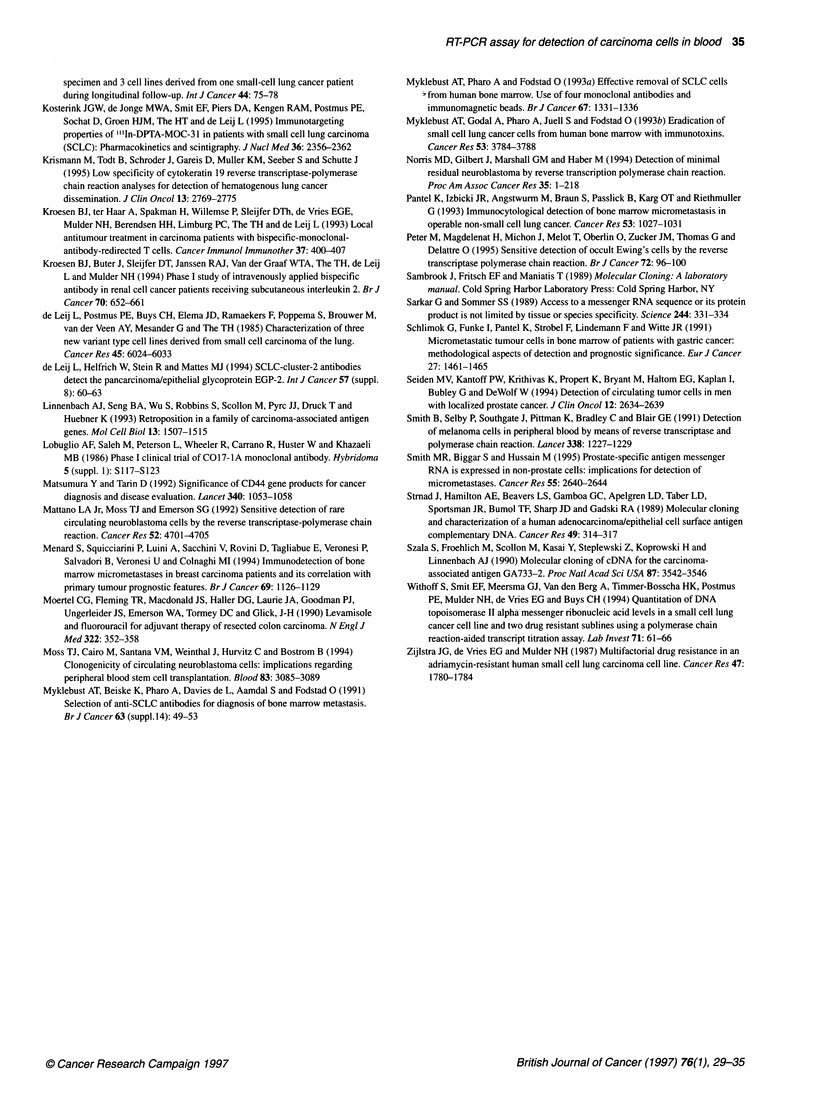# A quantitative reverse transcriptase polymerase chain reaction-based assay to detect carcinoma cells in peripheral blood.

**DOI:** 10.1038/bjc.1997.331

**Published:** 1997

**Authors:** W. Helfrich, R. ten Poele, G. J. Meersma, N. H. Mulder, E. G. de Vries, L. de Leij, E. F. Smit

**Affiliations:** Department of Clinical Immunology, University Hospital Groningen, The Netherlands.

## Abstract

**Images:**


British Journal of Cancer (1997) 76(1), 29–35
© 1997 Cancer Research Campaign

### A quantitative reverse transcriptase polymerase chain reaction-based assay to detect carcinoma cells in peripheral blood

**W Helfrich[1], R ten Poele[2], GJ Meersma[2], NH Mulder[2], EGE de Vries[2], L de Leij[1] and EF Smit[2,3]**

Departments of Clinical Immunology[1], Medical Oncology[2] and Pulmonary Diseases[3], University Hospital Groningen, Hanzeplein 1, 9713 GZ Groningen, The Netherlands

**Summary** The presence of tumour cells in the circulation may predict disease recurrence and metastasis. To improve on existing methods of cytological or immunocytological detection, we have developed a sensitive and quantitative technique for the detection of carcinoma cells in blood, using the reverse transcriptase polymerase chain reaction (RT-PCR) identifying transcripts of the pancarcinoma-associated tumour marker EGP-2 (KSA or 17-1A antigen). The amount of EGP2 mRNA was quantified using an internal recombinant competitor RNA standard with known concentration and which is both reversely transcribed and co-amplified in the same reaction, allowing for a reliable assessment of the initial amount of EGP2 mRNA in the sample. Calibration studies, seeding blood with MCF-7 breast carcinoma cells, showed that the assay can detect ten tumour cells among $1.0 \times 10^6$ leucocytes. The PCR assay revealed that normal bone marrow expresses low levels of EGP2 mRNA, although immunocytochemistry with the anti-EGP2 MAb MOC31 could not identify any positively stained cell. Analyses using this RT-PCR assay may prove to have applications to the assessment of circulating tumour cells in clinical samples.

**Keywords:** carcinoma; quantitative RT-PCR; metastasis; blood; bone marrow

Despite recent advances in cancer treatment, late metastatic disease continues to be a major problem in clinical management of malignancies, such as colorectal, lung, breast, and prostate carcinoma. Adjuvant therapy to reduce the risk of late recurrences appears to be beneficial in both breast and colorectal carcinoma (Fisher et al, 1989; Moertel et al, 1990). The detection of occult tumour cells in the peripheral blood of patients with solid tumours may be important in two different clinical situations. First, the finding of circulating tumour cells at diagnosis in patients with clinically localized disease responsive to chemotherapy, such as breast or colon cancer, might aid in the choice of adjuvant treatment options. Second, in the setting of autologous bone marrow transplantation (ABMT) or peripheral stem cell reinfusion after high-dose chemotherapy, contamination of the reinfused bone marrow or stem cell population with tumour cells might worsen prognosis (Anderson et al, 1989; Gribben et al, 1991; Brenner et al, 1993; Brugger et al, 1994; Moss et al, 1994). In recent years, especially in breast and colon cancer, it has been shown that occult contamination of the peripheral blood or bone marrow at diagnosis with tumour cells exerts an adverse influence on survival (Berger et al, 1988; Cote et al, 1991; Schlimok et al, 1991; Pantel et al, 1993; Diel et al, 1994; Harbeck et al, 1994; Menard et al, 1994). Often, in these studies, a monoclonal antibody (MAb) or a panel of MAbs directed against tumour cell-surface glycoproteins or cytokeratins was used to detect circulating tumour cells. The development of polymerase chain reaction (PCR)-based assays may allow a more sensitive tumour cell detection. Such assays have been described for a number of solid and haematological malignancies. EGP2 (also known as hEGP314, GA733-2 antigen, KSA, EGP40) is a 38-kDa transmembrane glycoprotein expressed on the surface of most simple epithelial cells and the majority of carcinomas such as colorectal, lung and breast carcinomas (de Leij et al, 1994). MAbs directed against EGP-2, such as CO17-1A, KS1/4 and MOC31, have been studied extensively in diagnostic and therapeutic approaches in cancer (Lobuglio et al, 1986; Elias et al, 1990; Kroesen et al, 1993; Herlyn et al, 1994; Kosterink et al, 1995). The human EGP2 cDNA was independently cloned by different research groups (Bumol et al, 1988; Strnad et al, 1989; Szala et al, 1990) and, more recently, its gene structure on chromosome 4q has been partly determined. Comparison of GA733-2 gene sequences with the previously established cDNA sequence revealed that this gene consists of nine exons interspersed by introns of variable length (Linnenbach et al, 1993).

In this report we describe the detection and quantitative analysis of very low amounts of EGP2 mRNA transcripts in bone marrow and peripheral blood by reverse transcriptase PCR (RT-PCR).

*Received 26 June 1996*
*Revised 2 January 1997*
*Accepted 13 January 1997*

*Correspondence to:* EF Smit, Department of Pulmonary Diseases, Free University Hospital, PO Box 7057, 1007 MB Amsterdam, The Netherlands

### MATERIALS AND METHODS

### Cell lines

The human small-cell lung cancer (SCLC) cell lines GLC-3, GLC-4 and GLC-14, the human breast carcinoma cell line MCF-7 and the human colorectal cancer cell line Colo 320 were selected as representatives of different types of carcinomas. SCLC cell lines GLC-3, GLC-4 and GLC-14 were chosen because of the different expression levels of EGP2 as determined by immunocytochemistry. All SCLC cell lines have been established in our laboratory and have been described previously (de Leij et al, 1985; Zijlstra et

**Figure 1** (**A**) Schematic representation of the human EGP2 gene. Shown are location of intron (–) and exon (□) sequences (adapted from Linnenbach et al) as well as the PCR (FW and REV) primers (solid arrows). Diagram is not to scale. (**B**) The construction of the EGP2 competitor RNA expression plasmid. A 198-bp *Bam*HI fragment was removed from plasmid CDM8EGP2 by digestion with *Bam*HI and subsequent plasmid religation yielding CDM8EGP2-B. Recombinant RNA was transcribed in vitro from the T7 promoter (open arrows). Amplification with the FW and REV primers gives rise to a product of 514 bp for normal EGP2 mRNA and 316 bp when using the recombinant competitor EGP2-B mRNA (solid bars). When genomic DNA is amplified no signal can be obtained, as the PCR primers span the EGP2 intron numbers to 3–6, constituting a DNA sequence of over 5 kb (see 1A). The restriction enzyme sites *Bam*HI and *Sca*I are indicated by B and S respectively. ○, Start codon; ●, Stop codon al, 1987; Berendsen et al, 1988). The MCF-7 and Colo 320 cell lines were obtained from the American Type Culture Collection (cat. nos HTB-22, CCL-220; ATCC Rockville, MD, USA). All cell lines were maintained in RPMI-1640 medium supplemented with 10% FCS, $5 \times 10^{-5}$ M β-mercaptoethanol and 1 mM sodium pyruvate, in a humidified 5% carbon dioxide atmosphere at 37°C.

### Collection of blood samples

Blood samples were taken from seven healthy volunteers after obtaining informed consent. All samples were collected in heparinized tubes on ice. In order to avoid contamination of blood cells with cells originating from the skin, which may contain some glandular cells expressing EGP2, at least two samples were drawn, of which the first was discarded.

### Bone marrow and peripheral stem cell harvests

Bone marrow was obtained from one of the seven healthy volunteers by aspiration. Additionally, bone marrow aspirates from three patients suffering from various haematological malignancies (chronic myelogenous and acute myelogenous leukaemias) were analysed. These bone marrow samples were chosen as a model for normal bone marrow as they are known to be negative for EGP2 in immunocytochemistry. Peripheral stem cell harvests from another three patients with haematological malignancies were collected. Again, these were chosen as a model for normal samples, i.e. not containing carcinoma cells.

### Immunocytochemistry

Cytocentrifuge slides of acetone-fixed cells were evaluated for EGP2 expression by incubation with the anti-EGP2 MAb MOC31 and subsequent indirect immunoperoxidase staining, according to standard procedures (Harlow and Lane, 1988). Endogenous peroxidase activity present in bone marrow and peripheral blood cells was inactivated by incubating the slides with 0.3% hydrogen peroxide for 5 min, after which the slides were rinsed three times with phosphate-buffered saline (9.0 mM disodium hydrogen phosphate, 1.3 mM sodium dihydrogen phosphate, 140 mM sodium chloride, pH 7.2) (PBS). Peroxidase-conjugated rabbit anti-mouse immunoglobulins were obtained from Dako (Glostrup, Denmark).

### RNA isolation

Large-scale isolation of total cellular RNA from cell lines was performed using the guanidine isothiocyanate–caesium chloride method (Chirgwin et al, 1979). Total cellular RNA from leucocytes and bone marrow was isolated using a modified acid phenol–guanidinium isothiocyanate method; peripheral blood was centrifuged for 10 min at 900 *g* and 4°C, and the plasma was removed. All subsequent steps were performed on ice and

© Cancer Research Campaign 1997

**Figure 2** Principle of the transcript titration assay. The assay is based on the fact that a mixture of two different EGP2 RNA templates, the normal EGP2 mRNA and the shorter 198-bp EGP2-B RNA, will compete for the same primers. Thus, when both RNA templates are present in a RT-PCR reaction mixture in equimolar amounts, they will be reversely transcribed and amplified accordingly. A titration series of competitor RAN solutions, decreasing in concentration, is mixed with a fixed amount of total cellular RNA containing the EGP2 mRNA to be quantified. After RT-PCR all subsequent reaction products of the titration series were analysed by gel electrophoresis. The relative intensity of the bands (514 bp vs 316 bp) per lane in determined by densitometric scanning. Lane 3 showing a relative intensity ratio of 1:1 for both bands is used to quantify the initial amout of EGP2 mRNA in the sample prechilled solutions were used. Erythrocytes were removed by lysis with ammonium chloride (155 mM ammonium chloride, 10 mM potassium bicarbonate, 0.1 mM sodium EDTA). Leucocytes were pelleted by centrifugation and washed with PBS. The cells were resuspended in 1 ml of PBS and counted. The cell suspension was transferred to a fresh microfuge tube, after which the cells were pelleted by centrifugation for 10 min at 900 ***g***. The cell pellet was dissolved in 500 μl of GITC (4 M guanidinium-isothiocyanate, 25 mM sodium citrate pH 7, 0.5% sarcosyl, 0.1 M β-mercapto-ethanol) by vigorous vortexing. Subsequently, 50 μl of sodium acetate (3 M, pH 5.0), 500 μl of water-saturated phenol and 100 μl of chloroform–isoamyl alcohol (49:1, v/v) were added and mixed well by vortexing. The mixture was left on ice for 10 min, after which it was centrifugated at 15 000 ***g*** and 4°C. The supernatant was transferred to a fresh microfuge tube, and precipitated by adding an equal volume of isopropanol and placing it at –20°C for 1 h. RNA was pelleted by centrifugation at 15 000 ***g*** and 4°C, and reprecipitated in 150 μl of GITC and isopropanol. RNA was pelleted by centrifugation as described above, and washed with 70% ethanol. The pellet was dried in a vacuum exicator and dissolved in 30 μl of diethylpyrocarbonate-treated water (DEPC, Sigma, St Louis, MO, USA). The integrity of the RNA samples was assessed on formaldehyde-containing (2.2 M) agarose gels. Only RNA samples with visible and discrete 28S and 18S ribo-somal RNA bands were used for the RT-PCR experiments.

### Design of the EGP2 RT-PCR

The human EGP-2 gene consists of nine exons interspersed by introns of variable length (Szala et al, 1990). EGP2-specific PCR primers were computer designed to span three introns and were subsequently synthesized using a commercial oligonucleotide synthesizer (Gene Assembler plus, Pharmacia, Uppsala, Sweden). The sequences of the EGP2-specific primers are: forward (FW): 5′-GAACAATGATGGGCTTTATG-3′ (corresponding to bases 374 to 394 of the EGP2 cDNA) and reverse (REV): 5′-TGAGAATTCAGGTGCTTTTT-3′ (bases 868–888). Amplification of EGP2 cDNA with these primers gives rise to a 514-bp product. When genomic DNA is amplified, no signal is obtained as the PCR primers span the EGP2 intron numbers 3–6, constituting a DNA sequence of at least 5 kb. A schematic representation of the human EGP2 gene (GA733–2) with location of the introns and exons, as well as the PCR (FW and REV) primers, is given in Figure 1A.

### Construction of an EGP2 competitor RNA expression plasmid

DNA manipulations were performed essentially according to Sambrook et al (1989). The plasmid CDM8 EGP2, encompassing the entire EGP2 cDNA, was a kind gift from Dr Linnenbach, The Wistar Institute, Philadelphia, PA and is described by Szala et al (1990). A 'deletion' competitor EGP2 cDNA construct was generated from this

© Cancer Research Campaign 1997

**Figure 3** Quantitative analysis using the EGP2 RT-PCR. A titration series of competitor RNA solutions was mixed with 100 ng of total cellular RNA derived from the EGP2-positive cell line MCF-7. Shown are the subsequent RT-PCR reaction products after gel electrophoresis/ethidium bromide staining. Lane M, DNA molecular weight marker VI (Boehringer Mannheim) – the upper band represents 653 bp; lane 1, no competitor RNA; lane 2,1 pg of competitor; lane 3, 5 pg; lane 4, 10 pg; lane 5, 25 pg; lane 6, 50 pg; lane 7, 75 pg; lane 8, 100 pg; lane 9, 250 pg competitor. The 316-bp PCR product derived from the competitor RNA (**B**) is readily distinguishable from the 514-bp product generated from the normal EGP2 mRNA transcript obtained (**A**). After densitometry the initial amount of EGP2 mRNA in the MCF-7 sample was calculated to be 18.9 pg 100 ng$^{-1}$ total cellular RNA

**Table 1** Mean (± s.d.) EGP2 expression levels in carcinoma cells lines calculated from the quantitative RT-PCR assays ($n$ = 3)

| Cell line | mRNA copy number per cell |
|---|---|
| GLC-3 | 650 (130) |
| GLC-4 | No expression detected |
| GLC-14 | 150 (40) |
| Colo 320 | 15 (5) |
| MCF-7 | 1500 (200, $n$ = 6) |

plasmid by digestion of the two *Bam*HI sites present within the EGP2 coding region, at positions 635 and 843. This procedure excises a 198-bp fragment from the EGP2 cDNA. After religation the plasmid was designated CDM8 EGP2-B. When subjected to PCR amplification with EGP2-specific primers (see above), CDM8 EGP2-B gives rise to a 316-bp product that is readily distinguishable from the 514-bp product generated from the normal EGP2 cDNA on a 1.5% agarose gel with ethidium bromide staining. A schematic outline of the construction procedures and the expected PCR products is given in Figure 1B.

### Synthesis of recombinant competitor RNA

All buffers and solutions were made RNAase free by treatment with DEPC. Recombinant competitor RNA was transcribed in vitro from the T7 promoter present in the vector located upstream of the EGP2-B insert, using the Ambion Megascript in vitro Transcription Kit according to the manufacturer's recommendations. For synthesis of run-off transcripts of defined size, CDM8 EGP2-B was linearized by digestion with *Sca*I. The resulting produced blunt ends avoid the generation of unwanted transcripts observed from 3′ overhanging ends. This transcription procedure gives rise to competitor RNA transcripts of 2015 bp. The cDNA template was specifically degraded by a 20-min incubation at room temperature with RNAase-free DNAase I supplied with the transcription kit. DNAase I was then inactivated by heating at 80°C for 5 min. Competitor RNA was subsequently purified using the caesium chloride centrifugation procedure, according to standard procedures (Chirgwin et al, 1979). The concentration of recombinant competitor RNA was determined spectrophotometrically.

### cDNA synthesis

An aliquot of either 100 ng (for cell lines) or 500 ng (for blood or bone marrow samples) of total cellular RNA was mixed with a variable amount of synthetic competitor RNA, ranging from 100 pg to 0.5 fg. To this was added 0.5 μl of RNAguard (Pharmacia) and 16.6 ng of EGP2 REV primer to a final volume of 9 μl. The EGP2 REV primer, used here as the first-strand primer, was allowed to anneal at 54°C for 30 min. Subsequently added were cDNA reaction buffer (Pharmacia), 8 U of MMuLV reverse transcriptase (Pharmacia) and another 0.5 μl of RNAguard to a total volume of 20 μl. The reaction mixture was incubated at 37°C for 90 min.

### Polymerase chain reaction conditions cDNA obtained in the previous step was subjected to 30 PCR cycles for cell line samples and 40 cycles for blood or bone marrow samples (denaturing at 94°C for 30 s, annealing at 54°C for 60s, elongation at 72°C for 90 s) using a DNA thermal cycler (Perkin Elmer Cetus, Norwalk, CT, USA). Before amplification all samples were subjected to an initial denaturation for 180 s. The final elongation step was extended by 10 min.

PCR reactions were performed with 0.125 U of *Thermus aquaticus* polymerase (Supertaq, HT Biotechnology, Cambridge, UK), in the reaction buffer supplied by the manufacturer, supplemented with 1.75 μM magnesium chloride, 200 μM dNTPs, and 300 ng of both FW and REV primers.

Optionally, a second-round PCR was performed using the same set of primers; the reaction products were diluted 1:2 and purified by phenol–chloroform extraction; 1 μl of the aqueous phase was used in a subsequent PCR reaction. False-positive signals were controlled for in this second round by using appropriate positive and negative controls.

### Quantitative RT-PCR procedure

The principle of the transcript titration assay used has been described previously (Kok et al, 1989; Withoff et al, 1994) and is schematically outlined in Figure 2.

### RESULTS

### Quantitative RT-PCR procedure

The results of a representative EGP2 quantitative RT-PCR assay are shown in Figure 3. An experiment is shown in which a titration series of competitor RNA solutions, ranging from 1 pg to 250 pg, is mixed with 100 ng of total cellular RNA from the EGP2-positive cell line MCF-7. After RT-PCR all subsequent reaction products of the titration series were analysed by gel electrophoresis (Figure 3, lanes 3–9). A 316-bp product derived from the competitor RNA was readily distinguishable from the 514-bp product generated from the normal EGP2 mRNA transcript. After densitometry, lane 6 was judged to display a relative intensity ratio of 1:1 for both bands. Consequently, this lane was used to quantify the initial amount of EGP2 mRNA in the sample, which was calculated to be 18.9 pg 100 ng$^{-1}$ total RNA.

© Cancer Research Campaign 1997

**Figure 4** Sensitivity of the EGP2 RT-PCR assay. The sensitivity of the assay was assessed by seeding peripheral blood with MCF-7 cells. After two rounds of PCR, ten MCF-7 cells in $1.0 \times 10^6$ leukocytes could still be visualized (lane 5). Lane M, molecular weight marker (DNA molecular weight marker VI, Boehringer Mannheim); lane 1, leucocytes; lane 2,1 MCF-7 cells/$1.0 \times 10^6$ leucocytes; lane 3, 5 MCF-7 cells / $1.0 \times 10^6$ leucocytes; lane 4,10 MCF-7 cells/$1.0 \times 10^6$ leucocytes; lane 5,100 MCF-7, cells/$1.0 \times 10^6$ leucocytes; lane 6, MCF-7 (positive control); lane 7, GLC4 (negative control); lane 8, competitor; lane 9, water blank

### EGP2 expression in human carcinoma cell lines

EGP2 expression was assessed by both immunocytochemistry and quantitative EGP2 RT-PCR in GLC-3, GLC-4, GLC-14, MCF-7 and Colo 320. All cell lines, except GLC-4, were found to be EGP-2 positive in both assays. The EGP2 expression levels in these cell lines calculated from the quantitative RT-PCR assays are given in Table 1. The calculation of the copy numbers per cell is according to methodology reported previously (Withoff et al, 1994).

### Sensitivity of the EGP2 RT-PCR assay

The sensitivity of the assay was assessed by seeding peripheral blood with MCF-7 cells. The MCF-7 cell line was chosen as it showed the highest expression of EGP2. After one round of PCR, ten MCF-7 cells in $1 \times 10^5$ leucocytes could still be visualized. Performing another round of PCR increased sensitivity to ten MCF-7 cells detectable in $1.0 \times 10^6$ PMN cells. The results of the sensitivity of the EGP2 RT-PCR assay are shown in Figure 4.

### EGP2 expression in peripheral blood

PBL samples from seven healthy volunteers were analysed for EGP2 expression by immunocytochemistry using the anti-EGP2 MAb MOC31. From each volunteer at least two slides, approximately $4 \times 10^4$ cells, were inspected. All seven samples were found to be negative. This observation was verified by performing two successive rounds of PCR with the EGP2-specific primers performed on total cellular RNA isolated from the same PBL samples. Again, using this assay no PCR product could be detected upon gel electrophoresis. Furthermore, the B-cell, T-cell and monocyte fractions, isolated by Ficoll–Hypaque from a pool of buffy coats, all proved to be EGP-2 negative in both immunocytochemistry with MAb MOC31 and the EGP2 RT-PCR assay (two rounds of amplification). Total cellular RNA isolated from GLC-3 was used as a positive control, whereas RNA isolated from GLC-4 was used as a negative control (data not shown).

### EGP2 expression in bone marrow and peripheral stem cell harvests

A bone marrow sample obtained from one of the seven healthy volunteers (see above) and three additional bone marrow samples obtained from patients with various haematological malignancies were analysed by both immunocytochemistry and the competitive RT-PCR assay. All the bone marrow samples proved to be negative in immunocytochemistry with the anti-EGP2 MAb MOC31. However, the EGP2 RT-PCR assay demonstrated a distinct 514-bp band of various intensity in all bone marrow samples analysed. Calculating from quantitative RT-PCR data, the expression of EGP2 in bone marrow appears to be very low, i.e. between 1 and 10 pg 5.0 $\mu g^{-1}$ of total cellular RNA. The identity of the detected band as a specific EGP2 PCR product was established by digestion with the restriction enzyme *Bam*HI (see *Bam*HI sites depicted in Figure 1B), which subsequently yielded three DNA fragments of the expected lengths (data not shown): 262 bp, 198 bp and 54 bp.

Peripheral stem cell harvests from another three patients with haematological malignancies subjected to EGP2 RT-PCR were also positive in the PCR assay (data not shown).

### DISCUSSION

The prognosis of patients with malignant tumours deteriorates with metastatic spread of disease. As therapeutic measures vary, correct assessment of tumour stage is essential clinical information. Commonly, the detection of carcinoma cells in the bone marrow and peripheral blood is based on morphology or on the immunological demonstration of proteins specific for epithelia or tissues using MAbs against the corresponding antigens, e.g. MAb MOC31 directed against the pancarcinoma-associated glycoprotein 2 (EGP-2) (Myklebust et al, 1991; Beiske et al, 1992; Myklebust et al, 1993*a*, *b*). The pancarcinoma-associated glycoprotein EGP2 is expressed in most epithelium-derived carcinomas and, as no expression of EGP-2 has been found in the peripheral blood of bone marrow using anti-EGP2 MAbs (Pantel et al, 1993), it was considered useful as a tumour marker for circulating tumour cells and bone marrow involvement. Thus, we assessed the possibility or detecting EGP-2-expressing cells (i.e. tumour cells) at the mRNA level by means of the sensitive polymerase chain reaction (RT-PCR). For a reliable estimation of the number of tumour cells involved, we measured the actual number of EGP2 mRNA transcripts using an internal recombinant competitor RNA standard with known concentration that is both reversely transcribed and co-amplified in the same reaction.

In this study we show that amplification by PCR with EGP2-specific primers on total cellular RNA isolated from the peripheral blood cell fraction of seven healthy volunteers was negative for EGP2 expression. Also, in isolated lymphocytes and monocytes no signal could be detected. The sensitivity of the assay was assessed by seeding peripheral blood with MCF-7 cells. After two rounds of PCR a minimum of ten MCF-7 cells in $1.0 \times 10^6$ leucocytes could be detected. This compares with techniques that rely on staining with MAbs when double-labelling techniques are used. Usually, the limit of detection with such a technique is one carcinoma cell per 4–5 $\times 10^5$ mononuclear cells. Data derived from several studies using RT-PCR techniques to identify carcinoma cells in peripheral blood samples show that the lower limit of detection is of the same order as found in this study (Smith et al, 1991; Matsumura and Tarin 1992; Mattano et al, 1992; Hardingham et al, 1993; Datta et

© Cancer Research Campaign 1997

al, 1994; Gerhard et al, 1994; Israeli et al, 1994; Seiden et al, 1994; Burchill et al, 1995; Peter et al, 1995).

Currently, the power of PCR technology seems to limit its use in clinical practice as it has been found that PCR methods can detect promiscuous transcription (Chelly et al, 1989; Sarkar and Sommer, 1989) of so-called tissue-specific genes. For example, PGP-9.5 (Norris et al, 1994), prostate-specific antigen (Smith et al, 1995), cytokeratin 19 (Krismann et al, 1995) and tyrosinase transcripts (Foss et al, 1995) can be amplified from peripheral blood of healthy volunteers. Illegitimate transcription of EGP2 may also be the explanation of the positive results obtained by us in bone marrow and peripheral stem cell harvests of patients suffering from various haematological malignancies. Alternatively, as staining with MAbs recognizing EGP2 is consistently negative in the bone marrow, it may be that EGP2 gene translation in the marrow results in a protein per cell level that is below the detection level of MAbs.

The quantitative approach described here may be able to deal with this problem as it enables us to define quantitatively an upper level of 'normal', namely illegitimate, transcription.

As it is logical to assume that the number of circulating tumour cells is an important parameter for patient outcome, a quantitative RT-PCR has obvious advantages. However, the results shown here indicate that considerable variation exists between tumour types as MCF-7 cells had a 100-fold higher EGP2 expression on level per cell than Colo 320 cells. Thus, the sensitivity of our assay might vary between and within different tumour types in vitro. Whether this phenomenon is present in vivo is not known. Should this prove to be the case, the copy number of EGP2 mRNA in cells of the primary tumour would have to be assessed before quantification of carcinoma cells in peripheral blood could be established.

In conclusion, we have developed a specific and sensitive quantitative RT-PCR assay to detect epithelium-derived carcinoma cells in peripheral blood. In bone marrow and peripheral stem cells, an unknown population of cells appears to express EGP2 mRNA, which makes this assay problematic for these specimens. The ability to detect very small numbers of carcinoma cells in the blood may provide the clinician with an important predictive tool with respect to recurrence and might help in a better selection for adjuvant therapy.

### ABBREVIATIONS

EGP-2, epithelial glycoprotein-2; MAb, monoclonal antibody; cDNA, complementary DNA; SCLC, small cell lung carcinoma; FCS, fetal calf serum; BSA, bovine serum albumin; bp, basepairs; kb, kilobasepairs; DEPC, diethylpyrocarbonate; PBS, phosphate-buffered saline (9.0 mM disodium hydrogen phosphate, 1.3 mM sodium dihydrogen phosphate, 140 mM sodium chloride, pH 7.2); RT-PCR, reverse transcriptase polymerase chain reaction; PBL, peripheral blood lymphocytes.

### REFERENCES

Anderson IC, Shpall EJ, Leslie DS, Nustad K, Ugelstad J, Peters WP and Bast RC Jr (1989) Elimination of malignant clonogenic breast cancer cells from human bone marrow. *Cancer Res* **49**: 4659–4664

Beiske K, Myklebust AT, Aamdal S, Langholm R, Jakobsen E and Fodstad O (1992) Detection of bone marrow metastases in small cell lung cancer patients. Comparison of immunologic and morphologic methods. *Am J Pathol* **141**: 531–538

Berendsen HH, de Leij L, de Vries EGE, Mesander G, Mulder NH, de Jong B, Buys CH, Postmus PE, Poppema S, Sluiter HJ and The TH (1988) Characterization of three small cell lung cancer cell lines established from one patient during longitudinal follow-up. *Cancer Res* **48**: 6891–6899

Berger U, Bettelheim R, Mansi JL, Easton D, Coombes RC and Neville AM (1988) The relationship between micrometastases in the bone marrow, histopathologic features of the primary tumor in breast cancer and prognosis. *Am J Clin Pathol* **90**: 1–6

Brenner MK, Rill DR, Moen RC, Krance RA, Mirro JJr and Anderson WFI (1993) Gene-marking to trace origin of relapse after autologous bone-marrow transplantation. *Lancet* **341**: 85–86

Brugger W, Bross KJ, Glatt M, Weber F, Mertelsmann R and Kanz L (1994) Mobilization of tumor cells and hematopoietic progenitor cells into peripheral blood of patients with solid tumors. *Blood* **83**: 636–640

Bumol TF, Marder P, DeHerdt SV, Borowitz MJ and Apelgren LD (1988) Characterization of the human tumor and normal tissue reactivity of the KS1/4 monoclonal antibody. *Hybridoma* **7**: 407–415

Burchill SA, Bradbury MF, Pittman K, Southgate J, Smith B and Selby P (1995) Detection of epithelial cancer cells in peripheral blood by reverse transcriptase-polymerase chain reaction. *Br J Cancer* **72**: 278–281

Chelly J, Concordet JP, Kaplan JC and Kahn A (1989) Illegitimate transcription: transcription of any gene in any cell type. *Proc Natl Acad Sci USA* **86**: 2617–2621

Chirgwin JM, Przybyla AE, MacDonald RJ and Rutter WJ (1979) Isolation of biologically active ribonucleic acid from sources enriched in ribonuclease. *Biochemistry* **18**: 5294–5299

Cote RJ, Rosen PP, Lesser ML, Old LJ and Osborne MP (1991) Prediction of early relapse in patients with operable breast cancer by detection of occult bone marrow micrometastases. *J Clin Oncol* **9**: 1749–1756

Datta YH, Adams PT, Drobyski WR, Ethier SP, Terry VH and Roth MS (1994) Sensitive detection of occult breast cancer by the reverse-transcriptase polymerase chain reaction. *J Clin Oncol* **12**: 475–482

Diel IJ, Kaufmann M, Costa SD and Bastert G (1994) Monoclonal antibodies to detect breast cancer cells in bone marrow. In *Important Advances in Oncology*. Hellman S and Rosenberg SA (eds). JB Lippincott: Philadelphia

Elias DJ, Hirschowitz L, Kline LE, Kroener JF, Dillman RO, Walker LE, Robb JA and Timms RM (1990) Phase I clinical comparative study of monoclonal antibody KS1/4 and KS1/4-methotrexate immunconjugate in patients with non-small cell lung carcinoma. *Cancer Res* **50**: 4154–4159

Fisher B, Costantino J, Redmond C, Poisson R, Bowman D, Couture J, Dimitrov NV, Wolmark N, Wickerham DL and Fisher ER (1989) A randomized clinical trial evaluating tamoxifen in the treatment of patients with node-negative breast cancer who have estrogen-receptor-positive tumors. *N Engl J Med* **320**: 479–484

Foss AJE, Guille MJ, Occleston NL, Hykin PG, Hungerford JL and Lightman S (1995) The detection of melanoma cells in peripheral blood by reverse transcription-polymerase chain reaction. *Br J Cancer* **72**: 155–159

Gerhard M, Juhl H, Kalthoff H, Schreiber HW, Wagener C and Neumaier M (1994) Specific detection of carcinoembryonic antigen-expressing tumor cells in bone marrow aspirates by polymerase chain reaction. *J Clin Oncol* **12**: 725–729

Gribben JG, Freedman AS, Neuberg D, Roy DC, Blake KW, Woo SDG, Rabinowe SN, Coral F and Freeman GJ (1991) Immunologic purging of marrow assessed by PCR before autologous bone marrow transplantation for B-cell lymphoma. *N Engl J Med* **325**: 1525–1533

Harbeck N, Untch M, Pache L and Eiermann W (1994) Tumour cell detection in the bone marrow of breast cancer patients at primary therapy: results of a 3-year median follow-up. *Br J Cancer* **69**: 566–571

Hardingham JE, Kotasek D, Farmer B, Butler RN, Mi JX and Sage RED (1993) Immunobead-PCR: a technique for the detection of circulating tumor cells using immunomagnetic beads and the polymerase chain reaction. *Cancer Res* **53**: 3455–3458

Harlow E and Lane D (1988) *Antibodies: A Laboratory Manual*. Cold Spring Harbor Laboratory Press: Cold Spring Harbor, NY

Herlyn D, Benden A, Kane M, Somasundaram R, Zaloudik J, Sperlagh M, Marks G, Hart E, Ralph C and Wettendorff M (1991) Anti-idiotype cancer vaccines: pre-clinical and clinical studies. *In Vivo* **5**: 615–623

Israeli RS, Miller WH Jr, Su SL, Powell CT, Fair WR, Samadi DSH, DeBlasio A, Edwards ET and Wise GJ (1994) Sensitive nested reverse transcription polymerase chain reaction detection of circulating prostatic tumor cells: comparison of prostate-specific membrane antigen and prostate-specific antigen-based assays. *Cancer Res* **54**: 6306–6310

Kok K, Osinga J, Schotanus DC, Berendsen HH, de Leij L and Buys CH (1989) Amplification and expression of different myc-family genes in a tumor

© Cancer Research Campaign 1997

specimen and 3 cell lines derived from one small-cell lung cancer patient during longitudinal follow-up. *Int J Cancer* **44**: 75–78

Kosterink JGW, de Jonge MWA, Smit EF, Piers DA, Kengen RAM, Postmus PE, Sochat D, Groen HJM, The HT and de Leij L (1995) Immunotargeting properties of $^{111}$In-DPTA-MOC-31 in patients with small cell lung carcinoma (SCLC): Pharmacokinetics and scintigraphy. *J Nucl Med* **36**: 2356–2362

Krismann M, Todt B, Schroder J, Gareis D, Muller KM, Seeber S and Schutte J (1995) Low specificity of cytokeratin 19 reverse transcriptase-polymerase chain reaction analyses for detection of hematogenous lung cancer dissemination. *J Clin Oncol* **13**: 2769–2775

Kroesen BJ, ter Haar A, Spakman H, Willemse P, Sleijfer DTh, de Vries EGE, Mulder NH, Berendsen HH, Limburg PC, The TH and de Leij L (1993) Local antitumour treatment in carcinoma patients with bispecific-monoclonal-antibody-redirected T cells. *Cancer Immunol Immunother* **37**: 400–407

Kroesen BJ, Buter J, Sleijfer DT, Janssen RAJ, Van der Graaf WTA, The TH, de Leij L and Mulder NH (1994) Phase I study of intravenously applied bispecific antibody in renal cell cancer patients receiving subcutaneous interleukin 2. *Br J Cancer* **70**: 652–661

de Leij L, Postmus PE, Buys CH, Elema JD, Ramaekers F, Poppema S, Brouwer M, van der Veen AY, Mesander G and The TH (1985) Characterization of three new variant type cell lines derived from small cell carcinoma of the lung. *Cancer Res* **45**: 6024–6033

de Leij L, Helfrich W, Stein R and Mattes MJ (1994) SCLC-cluster-2 antibodies detect the pancarcinoma/epithelial glycoprotein EGP-2. *Int J Cancer* **57** (suppl. 8): 60–63

Linnenbach AJ, Seng BA, Wu S, Robbins S, Scollon M, Pyrc JJ, Druck T and Huebner K (1993) Retroposition in a family of carcinoma-associated antigen genes. *Mol Cell Biol* **13**: 1507–1515

Lobuglio AF, Saleh M, Peterson L, Wheeler R, Carrano R, Huster W and Khazaeli MB (1986) Phase I clinical trial of CO17-1A monoclonal antibody. *Hybridoma* **5** (suppl. 1): S117–S123

Matsumura Y and Tarin D (1992) Significance of CD44 gene products for cancer diagnosis and disease evaluation. *Lancet* **340**: 1053–1058

Mattano LA Jr, Moss TJ and Emerson SG (1992) Sensitive detection of rare circulating neuroblastoma cells by the reverse transcriptase-polymerase chain reaction. *Cancer Res* **52**: 4701–4705

Menard S, Squicciarini P, Luini A, Sacchini V, Rovini D, Tagliabue E, Veronesi P, Salvadori B, Veronesi U and Colnaghi MI (1994) Immunodetection of bone marrow micrometastases in breast carcinoma patients and its correlation with primary tumour prognostic features. *Br J Cancer* **69**: 1126–1129

Moertel CG, Fleming TR, Macdonald JS, Haller DG, Laurie JA, Goodman PJ, Ungerleider JS, Emerson WA, Tormey DC and Glick, J-H (1990) Levamisole and fluorouracil for adjuvant therapy of resected colon carcinoma. *N Engl J Med* **322**: 352–358

Moss TJ, Cairo M, Santana VM, Weinthal J, Hurvitz C and Bostrom B (1994) Clonogenicity of circulating neuroblastoma cells: implications regarding peripheral blood stem cell transplantation. *Blood* **83**: 3085–3089

Myklebust AT, Beiske K, Pharo A, Davies de L, Aamdal S and Fodstad O (1991) Selection of anti-SCLC antibodies for diagnosis of bone marrow metastasis. *Br J Cancer* **63** (suppl.14): 49–53

Myklebust AT, Pharo A and Fodstad O (1993*a*) Effective removal of SCLC cells from human bone marrow. Use of four monoclonal antibodies and immunomagnetic beads. *Br J Cancer* **67**: 1331–1336

Myklebust AT, Godal A, Pharo A, Juell S and Fodstad O (1993*b*) Eradication of small cell lung cancer cells from human bone marrow with immunotoxins. *Cancer Res* **53**: 3784–3788

Norris MD, Gilbert J, Marshall GM and Haber M (1994) Detection of minimal residual neuroblastoma by reverse transcription polymerase chain reaction. *Proc Am Assoc Cancer Res* **35**: 1–218

Pantel K, Izbicki JR, Angstwurm M, Braun S, Passlick B, Karg OT and Riethmuller G (1993) Immunocytological detection of bone marrow micrometastasis in operable non-small cell lung cancer. *Cancer Res* **53**: 1027–1031

Peter M, Magdelenat H, Michon J, Melot T, Oberlin O, Zucker JM, Thomas G and Delattre O (1995) Sensitive detection of occult Ewing's cells by the reverse transcriptase polymerase chain reaction. *Br J Cancer* **72**: 96–100

Sambrook J, Fritsch EF and Maniatis T (1989) *Molecular Cloning: A laboratory manual*. Cold Spring Harbor Laboratory Press: Cold Spring Harbor, NY

Sarkar G and Sommer SS (1989) Access to a messenger RNA sequence or its protein product is not limited by tissue or species specificity. *Science* **244**: 331–334

Schlimok G, Funke I, Pantel K, Strobel F, Lindemann F and Witte JR (1991) Micrometastatic tumour cells in bone marrow of patients with gastric cancer: methodological aspects of detection and prognostic significance. *Eur J Cancer* 27: 1461–1465

Seiden MV, Kantoff PW, Krithivas K, Propert K, Bryant M, Haltom EG, Kaplan I, Bubley G and DeWolf W (1994) Detection of circulating tumor cells in men with localized prostate cancer. *J Clin Oncol* **12**: 2634–2639

Smith B, Selby P, Southgate J, Pittman K, Bradley C and Blair GE (1991) Detection of melanoma cells in peripheral blood by means of reverse transcriptase and polymerase chain reaction. *Lancet* **338**: 1227–1229

Smith MR, Biggar S and Hussain M (1995) Prostate-specific antigen messenger RNA is expressed in non-prostate cells: implications for detection of micrometastases. *Cancer Res* **55**: 2640–2644

Strnad J, Hamilton AE, Beavers LS, Gamboa GC, Apelgren LD, Taber LD, Sportsman JR, Bumol TF, Sharp JD and Gadski RA (1989) Molecular cloning and characterization of a human adenocarcinoma/epithelial cell surface antigen complementary DNA. *Cancer Res* **49**: 314–317

Szala S, Froehlich M, Scollon M, Kasai Y, Steplewski Z, Koprowski H and Linnenbach AJ (1990) Molecular cloning of cDNA for the carcinoma-associated antigen GA733–2. *Proc Natl Acad Sci USA* **87**: 3542–3546

Withoff S, Smit EF, Meersma GJ, Van den Berg A, Timmer-Bosscha HK, Postmus PE, Mulder NH, de Vries EG and Buys CH (1994) Quantitation of DNA topoisomerase II alpha messenger ribonucleic acid levels in a small cell lung cancer cell line and two drug resistant sublines using a polymerase chain reaction-aided transcript titration assay. *Lab Invest* **71**: 61–66

Zijlstra JG, de Vries EG and Mulder NH (1987) Multifactorial drug resistance in an adriamycin-resistant human small cell lung carcinoma cell line. *Cancer Res* **47**: 1780–1784

© Cancer Research Campaign 1997